# Collaborative governance and personal relationships for sustainability transformation in the textile sector

**DOI:** 10.1038/s41598-024-64373-1

**Published:** 2024-06-10

**Authors:** Felix Beyers

**Affiliations:** 1 Research Institute for Sustainability - Helmholtz Centre Potsdam (RIFS), Potsdam, Germany; 2 Institute for Sustainability Governance (INSUGO), School of Sustainability, Leuphana University Lüneburg, Lüneburg, Germany

**Keywords:** Relational sustainability, Textile transformation, Global partnerships, Inner transformation, Sustainable development goals, Environmental economics, Psychology and behaviour, Sustainability

## Abstract

This paper explores the potential for collaborative governance in the textile sector to act as a catalyst for sustainability transformation. The article originated from a 4-year research project examining a multi-stakeholder initiative (MSI), the German Partnership for Sustainable Textiles. It sheds light on the complex but interdependent connections between collaborative governance and personal relationships. While emphasising the role played by MSIs in creating important space for negotiating interests, it points towards the co-benefits of building relationships beyond stakeholder boundaries. Obstacles such as governance structures and the fragmentation of the governance landscape hinder opportunities for personal, political, and practical transformation. While highlighting the importance of private governance, it also stresses the role of state regulation in global economies, e.g. in the current debate on the EU Due Diligence Act. Finally, suggestions are made for designing governance spaces that support the development of social relationships while promoting transformation by ensuring the equal participation of stakeholders, employing learning and facilitation experts, and promoting joint decision-making processes.

## Introduction

A better understanding of how human efforts drive the processes of sustainability transformation is key to a more sustainable future. Defined by Patterson et al.^1^ as fundamental shifts in socio-technical-ecological systems, sustainability transformation has become prominent in societal and political debate^2^. Incremental change proves insufficient^3^ in times of super-wicked problems (e.g. climate change)^4^, prompting questions about the forms of change that are necessary and how to implement those changes to reach deliberate, ethical, and sustainable system transformation^5^. In response, there is an increasingly concerted global effort focused on tackling unsustainable practices through global agendas such as the United Nations sustainable development goals (SDGs)^6^. Notwithstanding, their effectiveness in bringing about fundamental change, as well as agreement regarding the underlying drivers and barriers to change, remain a matter of debate^7^.

Collaborative governance represents a political attempt to achieve sustainability transformation through joint ventures linking different representatives of interest groups. Inter-organisational networks and governance partnerships have increasingly evolved in the textile and fashion sector in recent decades^8^. According to the definition by Emerson et al.^9^ collaborative governance brings together stakeholders to achieve public goals that cannot be achieved through state regulatory traditions^10^. In the debate on governance that emerged in the early 2000s, O’Rourke^11^ argued that regulation was, and still is Ref.^12^, simply "outsourced" to non-state actors. Governance efforts often manifest themselves in formal or informal institutions^13^, whereby structures and processes are usually distinct and characterised by different objectives and goals^14^. Lange et al.^14^ formulated a meta-framework for collaborative modes of governance, basing their arguments on the work of Driessen et al.^15^. They distinguish between top-down state-centred modes of governance and bottom-up self-governance linking non-state actors on a continuum between two extremes. Public–private governance along interactive governance principles, such as multi-stakeholder partnerships, can be positioned within this continuum according to the differing structures, processes, and content (polity, politics, and policy).

Although the legitimacy of such initiatives derives from tangible governance outcomes, collaborative efforts often have intangible social effects. A simple example is social learning between actors, which can play a decisive role in transformative change^16,17^. Social learning generally means that individuals learn with and from each other while negotiating and discussing their diverse perspectives and interests to explore their inner values and worldviews^18,19^. From Donella Meadows’s leverage points perspective^20–22^, touching upon these inner dimensions (mindsets, values, and worldviews) is key for achieving transformation.

Given that the social implications of collaborative governance are significant for transformative change, a better understanding of these aspects is crucial. However, the occurrence of social relationships and social learning, or the development of personal or inter-personal relationships where learning may occur, is often ignored in governance literature and is lacking in governance activities that allow for personal opinions and perspectives to be compared and interwoven. Political negotiators who play a representative role in governance processes^23^ act on behalf of their institution and their political or interest group. They rarely share personal perspectives and are often not even perceived as people with individual perspectives and their own values and worldviews. However, sustainability scientists argue that addressing precisely these inner dimensions is crucial to build leverage for collaboration and joint action for transformative change^24^. Taking this idea further, we ask what role human relationships play in political negotiations. What if personal opinions, emotions, and feelings could be included in sustainable politics and governance? Ultimately, can the renegotiation of personal values and perspectives form the basis for collective and political action to progress sustainability transformation?

Consequently, by drawing on evidence from an empirical case study, this paper argues that social relationships (and more specifically personal and inter-personal relationships) are relevant in enriching the policy debate on sustainability transformation. The German Partnership for Sustainable Textiles (henceforth the Textiles Partnership) is a German interactive collaborative governance initiative established in 2015. Stakeholders from seven stakeholder groups were interviewed and brought together for a reflective focus group discussion. The MSI was initiated by the German government as a response to the Rana Plaza factory collapse in Bangladesh where more than 1000 people died. This empirical case embedded in a broader governance landscape forms the basis for the theoretical argument that social relationships can be a crucial impetus for change and can complement the state-centred regulations currently under debate and enforced through due diligence. This paper, therefore, fits the criteria of sustainability transformation research by highlighting how social bonds and personal relationships are crucial for bridging divides and creating the necessary framework conditions to collectively solve problems by incorporating different interests and worldviews. This work could also open opportunities for further research into the areas of personal relationships in other policy contexts and the interplay between differing governance modes to foster sustainability transformation.

Section “Sustainability transformation, collaborative governance, and social relationships” of this paper presents an overview of theoretical perspectives on sustainability transformation, collaborative governance, and social relationships. Subsequently, Section. “State-of-the-art in collaborative textile governance: a super-wicked landscape and its barriers to transformation” outlines the state-of-the-art of sustainable textile governance in Germany. Section “Emerging disruptions: personal relationships as transformative interventions” highlights interventions and moments of disruption, before the learning and ideas for transformative change are showcased in Section. “Learning from disruption: harmonising governance frameworks and promoting personal relationships”. Section “Discussion & conclusion” discusses the findings and makes policy recommendations to overcome barriers and reach solutions. Finally, Sect. “Empirics & methodology” presents insights from the empirics and methodology.

## Sustainability transformation, collaborative governance, and social relationships

Theories that aim to shed light on efforts to achieve sustainability transformation offer valuable insights into what kind of change is needed and where to intervene. Davelaar^25^ brings together diverse perspectives on sustainability transformation, enhancing our understanding of the issue. She draws from Argyris and Schön's^26^ theory of single-loop, double-loop, and triple-loop learning, distinguishing three types of change: incremental, reform, and transformative^27,28^. Incremental change involves applying existing logic to different contexts, remaining "inside the box". Reform operates "outside the box", recognising the need for policy or organisational restructuring within the current status quo. Transformative change challenges and redefines goals, introducing fundamental shifts in operational logic that had not been previously considered.

Regarding the question of where to change or intervene, Davelaar highlights the leverage points framework that considers the possible points of leverage for intervening in a system^21,22,29^. Another perspective from this angle is O'Brien and Signa’s^5^ distinction between the societal dimensions in which these changes manifest themselves. The authors describe the interlocking spheres of transformation: the practical, political, and personal spheres. In a similar way to the theory of leverage points, different parameters are the target of change, such as the practical or outcome spheres which comprise pragmatic change and visible policy decisions as well as measurable effects. The political sphere encompasses the economic, political, legal, social, and cultural systems of society, influencing the preconditions for practical change and revealing the power dynamics crucial for transformation. Lastly, the personal sphere comprises the individual and collective convictions, values, and worldviews that shape the diverse logics of action. These different spheres are intertwined and influence each other through complex and interdependent relationships.

**Figure 1:**
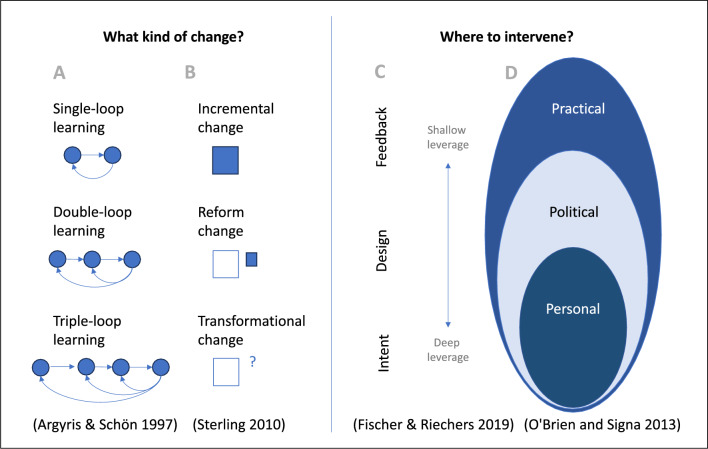
Shows a comparison of various theories related to systems and transformation. The left side of the figure differentiates between different types of change (**A**,**B**), while the right side indicates different intervention points or spheres for intervention (**C**,**D**). This comparison is based on Davelaar’s^25^ heuristic model combining the learning concepts of Agrys & Schön^26^, different types of change by Sterling^28^, Abson et al.’s^21^, as well as Fischer and Riechers’^22^ leverage points for sustainability transformation, and O'Brien and Signa’s^5^ spheres of transformation.

Figure 1 compares the different theories in terms of the types of change on the left (what kind of change?) and the intervention points on the right (where to intervene?). It highlights the various ways in which different aspects can be addressed, and how these approaches lead to different intensities in terms of outcomes. Overall, it illustrates that targeting mindsets and values results in the most profound outcomes for transformative learning, interventions, and change.

Drawing from the political literature, it is important to highlight theoretical perspectives on collaborative governance and political corporate social responsibility (PSCR). While governance lacks a single definition^30^, it generally denotes collective action within formal or informal systems involving public and/or private actors working toward common goals and "creating the conditions for ordered rule"^14,31^. Historically, governance evolved because non-state actors became involved in the processes and structures of state regulation due to regulatory gaps exposed by international trade agreements and intergovernmental organisations in increasingly liberal and globalised economies in the late twentieth century^15^. Corporations, perceived as major contributors to sustainability challenges, led the development of industry-based practices such as corporate social responsibility (CSR) and corporate sustainability^32,33^. CSR represents "the dramatic progress made by companies in recent decades in balancing shareholder goals with the need to reduce externalities that impact other stakeholders"^34^. Stakeholders, in this context, are those who can affect or be affected by a company's activities. They include customers, suppliers, shareholders, NGOs, media, and professional associations^35,36^. The demand for the adequate representation of all stakeholders gave rise to private governance arrangements^14^ and the establishment of new institutions (such as transnational sustainability governance associations) to facilitate deliberative decision-making between state and non-state actors^37^. Today, the field encompasses various collaborative governance modes within the framework of political CSR (PCSR)^38^, resembling a complex jigsaw puzzle^13,39^. However, the above-mentioned framework of modes of governance by Lange et al.^14,15^ helps us to distinguish five modes of governance with different structures, processes, and contents: centralised governance, decentralised governance, public–private governance, interactive governance, and self-governance.

A crucial element of collaborative governance is the interaction between stakeholders, characterised by modes of social interaction focused on gaining insight into how governance actors navigate the intricate governance landscape. Social interaction encompasses the behaviour of individual actors and their mutual exchanges. Eberlein et al.^40^ describe and emphasise the relevance of personal and social interactions in private governance contexts. They argue that inter-relationships and interactions between stakeholders represent the basis for comprehending the different effects on regulatory capacity, on performance, and on the social and environmental impacts (p. 1). They categorise interactions at the micro (personal) level into four types: competition, coordination, co-optation, and chaos. Competition denotes competitive interactions in the market and regulatory processes, while coordination signifies deliberate cooperation. Co-optation involves suppressive actions, and chaos refers to unpredictable and undirected interactions. An important aspect in the reflection of social relationships is that formal and informal formats always include a design question that enables interaction between stakeholders in governance. In this regard, a number of authors^41–43^ emphasise the need to design such a space to foster social interactions and personal relationships, where open discussions on values, emotions, and world views can take place to create trusting atmospheres alongside political negotiations. Therefore, design decisions are crucial, taking into account the overall objectives regarding the atmosphere, space, and methods for collaboration and joint action^44^.

Against this background, we now examine a case of interactive collaborative governance in the textile sector with its structures processes and social relationships, which is embedded in a country-specific governance landscape that encompasses the global economy with its super-wicked problems.

## State-of-the-art in collaborative textile governance: a super-wicked landscape and its barriers to transformation

The Textiles Partnership, launched in 2015, has played a significant role in German apparel and textile politics. It is an established MSI comprising seven stakeholder groups. With over 130 member organisations, it aims to support economic actors to voluntarily address sectoral risks and contribute to sustainability transformation through joint projects and learning. Although it has formed collaborations and alliances with other public and private initiatives, it has not yet achieved total buy-in from the industry. The industry participants account for around 50% of the German market share in the textile industry. However, there was a significant buy-in following intense negotiations surrounding the shift in 2017 from voluntary sustainability roadmaps to report on individual sustainability efforts to binding roadmaps. In addition, concrete working groups with selected stakeholder representatives on topic-specific sectoral risks were introduced. Previously, the events were open, resulting in varied groups and no clear direction. The MSI is part of the German government's broader textile strategy, which includes the National Action Plan on Business and Human Rights, the UN Due Diligence Guidance, the Green Button textile label, and the recently adopted Due Diligence Act. It is a noteworthy case of governance as it now spans over 9 years and is embedded in a dynamic governance landscape which is constantly evolving through government and stakeholder efforts.

In terms of the political sphere, the Textiles Partnership is embedded in a broader governance landscape, which is characterised by a large number of governance initiatives, each with its own structures, processes, and content^14^. The modes observed within the initiatives range from more centralised forms to public–private partnerships, multi-stakeholder initiatives, and informal self-governance constellations between civil society and industry^15^. This is reflected in the fact that the Textiles Partnership has 31 formal and informal collaborations with other initiatives, each of which has different practical implications. An example is the collaboration between the Textiles Partnership and the ACT initiative: "*It has been beneficial that some of the members of the Textiles Partnership have joined another, broader initiative. It's called Act and it says […] that the best wage is the one that is collectively negotiated, that you can go above and beyond what is paid as a statutory minimum wage in many countries—[…]"* (Interview (IT) 16, Union 01). One NGO representative agrees that a major advantage of the partnership is its ability to leverage individual networks and experiences: "*I think that this point of cooperation between the partnership and other initiatives [is] to use learning experiences that have already been made in well-selected international initiatives by individual companies and to carry them more broadly in the partnership*" (FGD, NGO 02). The empirical governance landscape thus corresponds to the idea that an increasing number of non-state actors are involved in activities that were traditionally state-centred^8^. It also supports the argument that private governance endeavours aim to establish new institutions^45^.

However, this diversity of governance initiatives and interactions poses challenges in the practical sphere of transformation. The different objectives of governance initiatives and how to achieve these initiatives expose companies to a variety of compliance mechanisms. Each initiative develops specific agendas and goals, leading to uncertainty about which strategy is most appropriate and timely for a business. Matters become even more complex when considering that governance initiatives also form partnerships, which then jointly define social and environmental standards. Since private forms of governance leave the regulation of sustainability practices to the market, many companies opt to keep their efforts low-profile. Others choose to introduce their sustainability projects or standards through small-scale ventures, which can lead to even greater fragmentation in the industry. Complying with regulations can be further complicated by the existence of both binding and non-binding agreements. As stated by one association member: "*I think the keyword "harmonisation" is particularly important for us from an economic point of view. […] To what extent does my involvement in the Textiles Partnership play a role for the Green Button and *vice versa*? […] why is it not possible to find recognition systems, […] for the members, in terms of reporting obligations?*" (FGD, Association 01).

In the personal sphere of transformation, this raises the question for sustainability managers of how and where they should become involved, given their limited resources and capacities. Which values and mindsets support which governance approach or initiative? Would it make better strategic sense to set in-house or independent standards? Decision-makers and representatives are, therefore, faced with an overly complex landscape and a multitude of governance initiatives with different compliance mechanisms that hinder decision-making and often lead to emotional paralysis. Not forgetting that there are also different interest groups within a company. Due to limited resources and a lack of coherence, resource-efficient decisions are often made. For example, companies opt for existing collaborations, such as the Textiles Partnership, but may not get actively involved. For example, we were able to show through a network analysis that only a few of the 130 organisations are active in the structures and processes of the Textiles Partnership, while many hardly engage in any activity (for more insights see Ref.^16^). This shows, that resources are rarely used optimally as the many efforts that are made at different levels generally fail to function harmoniously.

## Emerging disruptions: personal relationships as transformative interventions

Noteworthy interventions towards sustainability transformation occur within the personal sphere or the deeper leverage points, particularly in the context of the dynamics of building personal relationship beyond stakeholder groups. The interviews revealed that personal relationships evolved between NGO representatives and industry representatives. Both NGO representatives and industry players stated that the in-depth and long-term collaborative work (especially in the informal spaces) contributed to getting to know each other, which resulted in concrete sustainability efforts. One business stakeholder mentioned that “*[…] of course we have got to know each other well, and we also go on retreats once a year, where we sit together for two days overnight in a place where we are far away from work and can talk about other topics in the evening […], and I think that is very important*” (Transcript 09, Association 02). The Tamil Nadu project on the rights of women and workers is a good example of sustainability efforts that developed through the cooperation between various actors. In the initial phase of the project, industry representatives used their business connections with manufacturers to invite a delegation from the Textiles Partnership, which also included representatives from NGOs, to gain specific insights. Although only few members of the Textiles Partnership are involved in the Tamil Nadu project, the initiative "*has trained more than 10,000 workers in South India in their rights and has begun to build structures on the ground to support and encourage long-term dialogue on this issue*" (Transcript 04, Company 02). A company representative pointed out that intensive cooperation can lead to joint success: "*So afterward we always said: Would you ever have thought that [we] would sit down at a table and say quite clearly that what you think is nonsense and *vice versa*? And that we would manage to achieve something together in the end?*" (Transcript 03, Company 01).

This commitment from both sides contrasts with the historic mode of interaction of co-optation, where industry representatives did not collaborate with NGO representatives. NGOs focused primarily on advocacy and competitive strategies vis-à-vis market actors^46^. The Textiles Partnership case demonstrates a shift towards a collaborative and consultative approach with NGOs, which is now actively contributing to market regulation through shaping and content building. This change also occurred because individuals became explicitly involved and developed better relationships with each other through personal discussions – both formal and informal. Values and changing attitudes played an equally important role, as the personal perspectives that developed in inter-group processes in turn influenced intra-group decision-making. The purpose of communication shifted from criticism to building meaningful connections with industry stakeholders, indicating a departure from the traditional confrontational methods.

Considering the political sphere, a closer look at the governance structures of the Textiles Partnership illuminates how the initiative contributed to personal relationship-building as a transformative intervention. Positioned as an interactive governance initiative, the Textiles Partnership aims at social interaction and learning. However, the private governance conditions and the prioritisation of governance outcomes pose a challenge to comprehensive participation and limit the diversity of members who are involved in the governance processes. The first aspect refers to the fact that participation is voluntary and that the various organisations themselves decide to what extent they want to participate in governance. Small and medium-sized companies, for example, are often financially unable to hire employees for such sustainability roles and lack capacity to participate. By contrast, the second factor refers to external pressure, typically the questioning of the legitimacy of private initiatives; this leads to hasty decision-making and ignores the fact that bringing together diverse stakeholders needs time. Focusing on the Textiles Partnership, the structural shift in 2017 brought about polity changes that favoured and supported exchanges within smaller membership groups (inter-groups), including expert groups, partnership initiatives, and the steering committee. The deliberate reconfiguration in 2017 aimed to create spaces where actors from diverse stakeholder backgrounds could build trust and collaborate over extended periods, fostering better informed governance decisions^47^. The emphasis on these smaller and more focused groups facilitated in-depth engagement and relationship-building for some actors. However, this has resulted in unintended inequalities because not all members of the initiative are equally allowed to participate and active in contributing to the decision-making processes, as shown through the network analysis^16^. This demonstrates that structural governance decisions can have both advantages and disadvantages, an aspect which must be considered when formulating collaborative governance aims and goals.

In the case of the Textiles Partnership, structural change from previous topic-specific working groups to expert groups supported the practical implications and reform change represented in the practical sphere. The example of the roadmaps moving from voluntary to mandatory is significant. Roadmaps contain concrete risk assessments and corresponding recommendations along the value chain for action to bring about change on the ground. Thus, they are essentially the technical answers to the many sustainability challenges faced by the industry. The transition in status from the voluntary to the binding through political debate is an example of deliberative efforts. Industry players initially resisted the requirement. Grimm’s^48^ study on the Textiles Partnership examines why industry actors changed their perception of binding roadmaps, arguing that the concept changed from being “impossible” to “possible”, first by financial incentives and later through social incentives. This illustrates how it can be possible to achieve reform change through structures that favour governance outcomes and committed individuals who engage in intense policy debate.

## Learning from disruption: harmonising governance frameworks and promoting personal relationships

The harmonisation of governance mechanisms would help decision-making in the shallower leverage points and in the practical sphere. Interviewees stated that a key driver for transformational change is the harmonisation of different governance orders and rules. Strong, cross-border legal regulations need to be established at national and international levels to meet the challenges of sustainability in global markets. In this regard, it is important to emphasise the role played by the state (often inactive) in recent decades in global unsustainable economies. However, as an ever-increasing volume of governmental and supranational legislation is implemented to address global value chains in unsustainable economies, the question arises as to whether a new era has dawned. On the one hand, this is reflected in the fact that Germany has enacted a law on Due Diligence, but not in the fact that the government recently voted against the EU Due Diligence Regulation. However, there is at least a recognition that government actors in consumer countries must start by redefining their roles; they must move away from lazy and potentially interest-driven disengagement towards becoming facilitators of change and creating enabling framework conditions for sustainable markets. It is, however, still important to involve non-state actors in regulatory processes and to create sufficient space for constructive conflict and exchange for building personal relationships.

In summary, the Textiles Partnership's intensive efforts have helped to create legitimacy and to promote transformative change in the political arena. An important milestone in this regard is the recent entry into force of the Due Diligence Act in Germany in January 2023 and the final approval of the EU Sustainability Due Diligence Directive (CSDDD) in April 2024. It shall however accompany, rather than replace, private regulation. However, the fact that Germany recently voted against the EU Due Diligence Regulation showcases that transformative change by political processes is far from complete. In this context, deliberative approaches can provide support and shape discourses and actions that were not previously thought possible.

**Figure 2:**
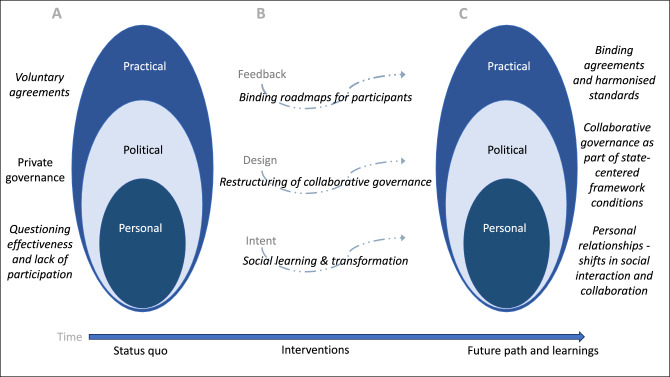
Visualisation of three different phases of the Textiles Partnership through O’Brien and Signa`s^5^ spheres of transformation with interventions^22^ as well as a timeline at the bottom of the diagram. Descriptions show how it was (**A**), what has happened (**B**), and where it should lead (**C**).

On a personal level, this means that developing social connections and establishing personal relationships can contribute to transformative change. Collaborative governance initiatives can emphasise the importance of building trust through social interaction and learning across stakeholder boundaries. Social learning is described as a transformative process that can help create systemic change to address sustainability challenges. However, it remains difficult to fully understand. In my interpretation, it is about the clash and friction between opinions, worldviews, and interests. This requires individuals to step out of their comfort zones, share informal moments, and to be open to learning and changing their perspectives. Figure 2 illustrates the different phases of the Textiles Partnership to illustrate the necessary action for deeper or transformative change.

## Discussion & conclusion

In a nutshell, this work provides empirical evidence that space for social interaction and learning in interactive collaborative governance can offer the necessary environment to bring mindsets and worldviews together to create genuine transformative potential. Conflicts are not necessarily resolved in these processes but instead form a bridge to negotiate worldviews and value systems and, ironically, to build social relationships between the former adversaries. Formal, but above all informal spaces offer the potential to facilitate and support relationships across the boundaries of stakeholder groups, which helps to bridge divides and promote joint learning and action. Personal relationships are thus a crucial component in collaborative governance for sustainability transformation. However, interactive governance is only one piece of the jigsaw in the wider context of governing global value chains. The diversity of political systems increases the complexity associated with the governance modes and decisions for regulating the global political economy. Governments must therefore question and redefine their role in the realm of governing global value chains.

In terms of the regulatory landscape, the legitimacy of collaborative governance has long been a subject of debate. Certain academics have traditionally questioned whether its proliferation signifies a failure of the state^49^ or emphasises deliberate structures^50^ to fulfil their task of ensuring the well-being of the population. Despite the pivotal function of the state in safeguarding public welfare, there is little empirical evidence in the literature of fostering innovative solutions through governments in global economies for the common good. This dearth of discourse on state regulation and its role in governance has been underscored by the transition from intergovernmental trade agreements to private, partnership-based, non-binding rule-making organisations within global supply chains in recent decades^8^. Critics argue that nations have increasingly deferred regulatory responsibilities to non-state actors^11^, contending that this shift was not merely the result of negligence but was intricately linked to the aim of making a profit^50^ in a capitalist economic paradigm^39^. The private sector, motivated by a desire to circumvent government regulation, has taken on governance functions. Through practices such as greenwashing, these have potentially exacerbated societal issues and deflected attention from underlying systemic problems. However, initiatives advocating for social change do exist amid these dynamics, indicating a nuanced landscape within collaborative governance^51^. Looking at experiences demonstrating the limitations of private regulation, voices calling for state intervention are gaining traction across various stakeholder groups and in the political discourse. This trend is evident in Germany and the EU, where there is a growing demand for state regulation not only from civil society but also from prominent market players and conservative industry associations. This evolving perspective recognises the need for national and international legislation to establish uniform standards and regulations across borders, thereby reasserting the state's regulatory role. Consequently, the EU decision on the Due Diligence Act is poised to play a critical role in the harmonisation of regulations.

Reflecting on personal relationships, this research paper indicates that the trajectory toward a shared understanding of the need for state legislation may have been facilitated in Germany through the extensive collaboration of diverse stakeholders within the Textiles Partnership^13^. This aligns with the contention that deliberative processes can engender a “meaning-making” endeavour that reshapes individuals’ core norms and perspectives. The intensive deliberative efforts of the partnership have notably transformed the relationship dynamics between NGOs and business representatives, shifting from a confrontational stance to one of collaboration and mutual engagement. This underscores the notion that spaces for social learning and interaction can be designed to foster and support changes in beliefs, values, and worldviews that can shape the range of feasible actions and strategies in practical settings. Rosenberg’s^52^ exploration of sustainability transformation among coffee farmers in Burundi further elucidates the significance of values in driving behavioural change, emphasising their inherent connection to daily practices. The Textiles Partnership case study has demonstrated how erstwhile adversaries have forged alliances through extensive interaction and participation in social learning spaces^16^, fostering trust even in informal settings. Thus, the research underscores the value of such initiatives in informing state legislation and fostering a collective recognition of the imperative for transformative measures in the textile industry. However, it is crucial to acknowledge that while these initiatives contribute to shaping legislative discourse and fostering a shared understanding of the need for transformation in the textile sector, their impact lies in complementing, rather than supplanting, impending legislation^13^.

In conclusion, I argue that interactive collaborative governance partnerships have an impact on transformation processes, but that they are limited by the need for stronger state regulation. The dichotomy between public and private governance is not a binary choice but rather a dynamic interplay from a meta-governance standpoint, as these modes can mutually reinforce each other. This viewpoint resonates with the findings of Lange et al.^53^, which suggest that non-hierarchical forms of governance must be considered alongside traditional structures for effective transformation. Thus, MSIs can contribute significantly to achieving sustainable and transformative outcomes but their efficacy in the textile sector hinges on strengthened state regulation, coordinated efforts at both international and European levels, the harmonisation of multiple governance initiatives, and the promotion of learning spaces with strategic design considerations (see Appendix A). To overcome these limitations, a variety of efforts must be made to ensure that nations start to assume their prescribed roles. They must debate, argue, and negotiate with other nations to foster harmonised efforts on a global scale, and simultaneously to create the necessary framework conditions in their sphere of influence in which actors can engage in collaborative governance. Only in this way can stakeholder participation, joint commitment, and harmonised efforts to solve the super-wicked problems be assured. For interactive governance modes, such as MSIs, equal participation of all interest groups is crucial to reflect the diversity of stakeholders and to ensure a wide variety but equal distribution of worldviews and interests. Against this background, the involvement of learning and facilitation experts is critical for gathering the necessary expertise and know-how to carefully design and host learning spaces that enrich the debate and foster the creation of personal bonds. Explorative design and facilitation efforts currently exist; for example, in the context of the UN climate negotiations where there is space to foster co-creative dialogues and reflection^42,43^. This research collective focuses on practices and principles to initiate new forms of dialogue in climate governance^41^. Such participatory formats reflect people's beliefs, values, worldviews, emotions, and motivations to create atmospheres of trust and authenticity. (For further insights into methods and principles specifically on mindsets, toolsets, and skillsets for co-creative dialogue and reflection, see Fraude et al.^42^). Nevertheless, the efficacy of governance partnerships cannot be measured solely by the creation of learning environments: they must also yield tangible governance outcomes. This duality necessitates a careful balancing act, wherein structures for governance decisions must involve a broad spectrum of stakeholders. Without this symbiosis, the transformative potential of these partnerships may remain confined to a subset of engaged individuals, failing to resonate with broader political and market interests. Therefore, effective coordination at both international and European levels is imperative, particularly given the transnational nature of the textile sector.

Further research in this field could include the exploration of the interplay between differing governance modes for sustainability transformation, as well as investigation into personal relationships in other policy contexts. For the former, further study into the alignment and interplay between different governance modes in the broader governance landscapes is needed. A better understanding of collaborative governance approaches in the context of state regulatory mechanisms and their mutual support is relevant. Exploring the interplay between public and private governance initiatives could provide insights into the barriers and synergies that may arise. Additionally, further case studies from the sector and beyond, examining different modes of governance, could provide valuable insights into how personal relationships can help or hinder governance for sustainability transformation. We recommend that future work explores collaborative governance modes to critically examine their role (and associated impacts) in shaping public policy and regulations for sustainability. This is where interdisciplinary and transdisciplinary methods and explorative transformative research can add value through holistic systems thinking and co-creative and participatory research methodologies. This ensures a better understanding of social interactions and exploration with co-creative and participatory principles, formats, and methods that help to better understand how research, participation, and policy design can impact sustainability transformation. In this way, sustainability science can use transformative research to help create knowledge and action for collaborative change.

## Empirics & methodology

The empirical basis for this conceptual contribution is the German textile sector with its complex governance landscape, and specifically the Textiles Partnership as a multi-stakeholder initiative (MSI). The textile sector has experienced remarkable growth on a global scale, witnessing a doubling of production between 2000 and 2015. Germany, as a prominent hub for textile production and consumption, is among the largest consumers in Central Europe, generating an impressive turnover of €11 billion in 2018. Reflecting the country's strong textile culture, the average German possesses approximately 95 clothing items and allocates around €78 per month for textiles and shoes. In 2018, Germany imported 1.4 million tonnes of clothing, primarily sourced from China, Bangladesh, and Turkey. This historical context highlights the sector's evolution from the emergence of sewing machines in 18th-century Great Britain to the current phenomenon of multinational outsourcing driven by labour-intensive processes. However, this expansion of global textile supply chains has also given rise to intricate sustainability challenges, ranging from environmental concerns to labour rights issues. These problems are further exacerbated by the prevailing trends of fast fashion and overconsumption, leading to the emergence of complex ethical dilemmas categorised as "super-wicked problems."

In response to these challenges, Germany underwent a transformative shift in its textile governance following the devastating Rana Plaza collapse in 2013. The government implemented various policies to address the industry's vulnerabilities, including the establishment of the Textiles Partnership in 2015. This multi-stakeholder initiative aimed to tackle sectoral risks and promote voluntary sustainability transformation. By 2021, the Textiles Partnership boasted a membership of over 130 organisations. However, it is worth noting that industry-wide buy-in remains below 50% by share. Despite this, the Textiles Partnership aligns with international standards, including the UN Due Diligence Guidance, contributing to the fulfilment of human rights obligations. Embedded in a dynamic governance landscape, the partnership forms alliances with various governance initiatives, showcasing the commitment of individual members and the effectiveness of multi-stakeholder governance. This nine-year long governance case, with its extensive membership plays a pivotal role in understanding the ongoing developments in the governance of the textile sector.

Empirically, this study employed a mixed-methods approach, combining both quantitative and qualitative data collection and analysis methods. The data collection process involved the use of academic literature and policy documents, complemented by 22 qualitative semi-structured interviews and a focus group discussion with members of the Textiles Partnership, serving as a single case study (see Appendix B). The choice of a critical case study approach was guided by its strategic importance in addressing the overall research problem^54^. The research was approved by the Leuphana Ethics Committee. All experiments were conducted according to Leuphana University guidelines and regulations, and informed consent was obtained from all participants.

For data analysis, several techniques and tools were employed. R Statistical Software (Version R 3.5.3 March 2019)^55^ was used for quantitative data analysis. Word cluster analysis, following the methodology by Abson et al.^56^, was conducted to extract meaningful patterns and relationships. Network analysis was performed using the igraph package^57^, allowing for the exploration of connections and interactions within the data. Additionally, qualitative content analysis was employed to analyse different document types and interview transcripts using MAXQDA by VERBI software^58^. By employing this comprehensive range of data collection and analysis methods, the research aimed to provide a holistic understanding of the textile sector and the governance dynamics associated with the Textiles Partnership.

### Supplementary Information

Supplementary Tables.

## Data Availability

The datasets generated and/or analysed for the current study are available from the corresponding author upon reasonable request.
