# Supplementary material for: Collaborative governance and personal relationships for sustainability transformation in the textile sector
Source: Sci Rep. 2024 Jun 10;14:13347. doi: 10.1038/s41598-024-64373-1 (PMC11164962; doi:10.1038/s41598-024-64373-1)
Supplement: Supplementary file 1 — Supplementary Tables. [file 41598_2024_64373_MOESM1_ESM.docx]

Appendices

Appendix A. Policy Brief

**Table A1.** Policy briefs for the effectiveness of Multi-stakeholder initiatives

| **Policy Brief:**  Enhancing Effectiveness of Multi-Stakeholder Initiatives (MSIs) in the Textile Sector  Summary: Multi-stakeholder initiatives (MSIs) play a crucial role in fostering collaboration among diverse stakeholders in the textile sector. To maximize their impact, certain key strategies and considerations must be addressed.  *Promoting Learning Spaces:*   - MSIs should actively cultivate learning spaces by integrating diversity into social interactions. - Encourage situations where members can build relationships, and trust, and resolve conflicts constructively. - Establish appropriate learning environments that push participants beyond their comfort zones.   *Focus on Design and Promotion:*   - Prioritize the design and promotion of effective learning spaces within MSIs. - Involve experts with extensive experience in the intricate field of collaborative learning. - Ensure that learning processes are well-structured and contribute meaningfully to the objectives of the MSIs.   *Strengthening State Regulation:*   - MSIs can achieve greater impact with stronger enabling state regulation. - Complement the legal framework by engaging in targeted collaboration with committed governance actors. - Seek to integrate participatory expert knowledge from non-state actors within a well-defined regulatory framework.   *Harmonization of Governance Initiatives:*   - Harmonize diverse governance initiatives falling under international frameworks. - Facilitate cooperation to bring about comprehensive change at multiple levels within the textile sector. - Address the challenge of fragmented efforts by aligning MSIs with overarching international standards.   *Coordination at International and European Levels:*   - Recognize the transnational nature of the textile industry and the importance of involving actors from producer countries. - Advocate for coordination of interactions at both international and European levels. - Ensure that formal structures of collaborative governance incorporate perspectives and contributions from diverse actors.   In conclusion, the effectiveness of MSIs in the textile sector hinges on the promotion of learning spaces, strategic design considerations, strengthened state regulation, harmonization of governance initiatives, and coordinated efforts at both international and European levels. By addressing these key aspects, MSIs can contribute significantly to achieving sustainable and transformative outcomes in the textile industry. |
| --- |

Appendix B. Empirical Data Overview

**Table A2.** Online sources downloaded for analysis: https://www.textilbuendnis.com/downloads/

| **Nr.** | **Document** | **Publication Date** | **Reference** |
| --- | --- | --- | --- |
| 1 | Action plan: Partnership for Sustainable Textiles | April 2015 | Textiles Partnership, 2015 |
| 2 | Rules of Interaction: Partnership for Sustainable Textiles | January 2018 | Textiles Partnership, 2018 A |
| 3 | Annual Report 2017 | November 2017 | Textiles Partnership, 2017 |
| 4 | Annual Report 2018 | November 2018 | Textiles Partnership, 2018 B |
| 5 | Annual Report 2019 | November 2019 | Textiles Partnership, 2019 |
| 6 | Response of the Partnership for Sustainable Textiles to the OECD Alignment Assessment | February 2020 | Textiles Partnership, 2020 |

**Table A3.** Interviewee list and focus group discussion separated by the different groups of actors

| **Stakeholder** | **Interview transcripts** | **Focus group discussion** |
| --- | --- | --- |
| State | 2 | 1 |
| Company | 5 | - |
| Association | 2 | 1 |
| NGO | 6 | 2 |
| Union | 1 | - |
| Standards organisation | 2, 1 written response | 1 |
| Advisory member | 3 | - |
